# Pharmacokinetics and pharmacodynamics of valganciclovir in infants with severe HIV-associated pneumonia in Africa: a sub-study of the EMPIRICAL randomised controlled trial

**DOI:** 10.1016/j.eclinm.2026.103899

**Published:** 2026-04-13

**Authors:** Tom G. Jacobs, Vivian Mumbiro, John Tembo, Franklyn N. Egbe, Cinta Moraleda, Laize Sílvia dos Anjos Botas Beca, Alfeu Passanduca, Lawrence Kakooza, Moses Chitsamatanga, Bwendo Nduna, Justina Bramugy, Alfredo Tagarro, Kajal D. Chhaganlal, Sophie Mutesi, Nancy L. Mark, Mutsa Bwakura-Dangarembizi, Natasha Namuzizya, Alvaro Ballesteros, Sara Dominguez-Rodríguez, Angela Colbers, Jahit Sacarlal, Damalie Nalwanga, Hilda A. Mujuru, Chishala Chabala, Lola Madrid, W. Chris Buck, Victor Musiime, Matthew Bates, Pablo Rojo, David M. Burger, EMPIRICAL Clinical Trial Group, Uneisse Cassia, Muhammad Sidat, Elias Manjate, Sónia Martins, Stella Langa, Natália Nipaco, Sara Machava, Anastância Chirindza, Luzidina Martins, Mércia Nhaca, Sílvia Chaúque, Dalila Rego, Dália Machel, Amir Seni, Celda Mavume, Belinda Macmillan, Aurora Mucarenga, Adelina Manheche, Edson Arcanjo, Maria Teresa Gatoma, Merunissa Gafur, Atija Salimo, Raul Atibo, Hélio Brande, Rito Eleutério, Marizelina Chaves, Atija Salimo, Armando Saíde, Kusum J. Nathoo, Moses Chitsamatanga, Ruth Marange, Shepherd Mudzingwa, Dorothy Murungu, Natasha Namuziya, Idah Zulu, Perfect Shankalala, Mulima Mukubesa, Juliet Namwinwa, Chalwe Chibuye, Terence Chipoya, Veronica Mulenga, Bwalya Simunyola, John Tembo, Muleya Inambao, Salome Chitondo, Wyclef Mumba, Endreen Mankushe, Henry Musukwa, Davies Sondashi, Albert Kamugisha, Karen Econi, Andrew Kiggwe, Judith Beinomugisha, Sharafat Nkinzi, Lawrence Kakooza, Henriator Namisanvu, Nancy Lajara Mark, Josam Thembo Mwesige, Ivan Segawa, Joseph Ssessanga, Paul Mbavu, Bosco Kafufu, Denis Nansera, Elizabeth Najjingo, Bashira T. Mbabazi, Abbas Lugemwa, Mariam Kasozi, Rogers Ankunda, Lilit Manukyan

**Affiliations:** a Department of Pharmacy, Pharmacology, and Toxicology, Radboud University Medical Center, Nijmegen, the Netherlands; b Department of Pharmacy, Tergooi Medical Center, Hilversum, the Netherlands; c University of Zimbabwe Clinical Research Centre, Harare, Zimbabwe; d HerpeZ Infection Research and Training, University Teaching Hospital, Lusaka, Zambia; e Clinical Infection, Microbiology, and Immunology, Institute of Infection, Veterinary and Ecological Sciences, University of Liverpool, UK; f Instituto de Investigación Sanitaria Hospital 12 de Octubre (imas12)-Fundación Biomédica del Hospital Universitario 12 de Octubre (FIB-H12O) Madrid, Spain; g Pediatric Service, Hospital Universitario 12 de Octubre, Servicio Madrileño de Salud, Madrid, Spain; h Department of Pharmacy, Lúrio University, Nampula, Mozambique; i Universidade Eduardo Mondlane Faculdade de Medicina, Maputo, Mozambique; j Makerere University Lung Institute, Kampala, Uganda; k Arthur Davidson Children's Hospital, Ndola, Zambia; l Centro de Investigação em Saúde de Manhiça (CISM), Maputo, Mozambique; m Pediatric Service, Infanta Sofia University Hospital, Servicio Madrileño de Salud, Madrid, Spain; n Universidad Europea de Madrid, Madrid, Spain; o Department of Research, Faculty of Health Sciences, Universidade Catolica de Mocambique, Beira, Mozambique; p Department of Paediatrics and Child Health, School of Medicine, College of Health Sciences, Makerere University, Kampala, Uganda; q University Teaching Hospitals-Children's Hospital, Lusaka, Zambia; r School of Medicine, University of Zambia, Lusaka, Zambia; s London School of Hygiene and Tropical Medicine, London, United Kingdom; t University of California Los Angeles David Geffen School of Medicine, Los Angeles, CA, USA; u Joint Clinical Research Centre, Kampala, Uganda; v School of Natural Sciences, University of Lincoln, Lincoln, United Kingdom; w Complutense University of Madrid, Madrid, Spain

**Keywords:** Infants, Pneumonia, Cytomegalovirus, HIV, Pharmacokinetics

## Abstract

**Background:**

Cytomegalovirus (CMV) is a major unrecognised cause of morbidity and mortality in infants living with HIV. Valganciclovir, an oral prodrug of ganciclovir, treats CMV in immunocompromised patients, but pharmacokinetic data in infants living with HIV are lacking. This study evaluated exploratory valganciclovir pharmacokinetic and pharmacodynamic outcomes in infants with severe HIV-associated pneumonia.

**Methods:**

As part of the EMPIRICAL clinical trial (ClinicalTrials.gov: NCT03915366, recruitment closed), infants living with HIV aged 1–12 months received valganciclovir 16 mg/kg twice daily. Pharmacokinetic sampling occurred on day 3 after enrolment at 2- and 5 h post-morning dose. Plasma CMV viral load was measured on days 0 and 15. The area-under–the-curve for ganciclovir over a 12-h interval (AUC_0–12 h_) was estimated using a limited sampling equation. Geometric mean AUC_0–12 h_ and the proportion of participants within the adult AUC_0–12 h_ target range (40–60 h mg/L) were calculated. Associations of ganciclovir AUC_0–12 h_ with covariates and log-change in plasma CMV viraemia were evaluated using linear regression. Infants were enrolled in this study between August 2020 and August 2022.

**Findings:**

Of 98 participants, 87 had evaluable pharmacokinetic profiles. Geometric mean AUC_0–12h_ (%CV) was 38.5 (54.8) h·mg/L. Only 35% achieved the adult AUC_0–12 h_ target; 47% were below, and 18% above it. Reduced renal function was the only covariate associated with higher ganciclovir AUC_0–12 h_. Ganciclovir AUC_0–12 h_ did not correlate with plasma CMV viral load reduction.

**Interpretation:**

Approximately two-thirds of infants was not within adult pharmacokinetic targets at 16 mg/kg/dose of valganciclovir. However, ganciclovir exposure was not predictive for virologic response or toxicity, suggesting lower exposures did not affect treatment efficacy in the EMPIRICAL trial.

**Funding:**

This project is funded by the European and Developing Countries Clinical Trials Partnership (EDCTP2) program supported by the 10.13039/501100000780European Union (RIA2017MC-2013).

**Research in context**

**Evidence before this study**

We reviewed the available evidence on valganciclovir and ganciclovir pharmacokinetics and pharmacodynamics in infants and young children. We searched PubMed and Web of Science for studies published up to 2022, without language restrictions. Search terms included combinations of “cytomegalovirus”, “CMV”, “valganciclovir”, “ganciclovir”, “pharmacokinetics”, “AUC”, “infants”, “children”, and “HIV”. We also screened reference lists of relevant reviews and clinical trials.

Most available valganciclovir and ganciclovir pharmacokinetic data originated from children treated for congenital CMV infection or from paediatric solid organ transplant recipients receiving CMV prophylaxis or treatment. These studies were generally small and heterogeneous with respect to age and dosing strategies. In congenital CMV studies, infants were often younger than 1 month of age, whereas studies in paediatric solid organ transplant recipients predominantly included children older than 1 year.

The ganciclovir exposure-response target (AUC_0–12 h_ 40–60 mg∗h/L) based on adult populations was commonly applied to children. Previous paediatric studies showed that many children receiving valganciclovir at a dose of 16 mg/kg had ganciclovir exposures below the adult target. However, none of these studies confirmed appropriateness of this target. No pharmacokinetic studies were identified that evaluated oral valganciclovir in infants with severe HIV-associated pneumonia, assessed pharmacokinetic–pharmacodynamic relationships for postnatal CMV disease in this population, or included children from Africa. No pooled meta-analyses of optimal ganciclovir exposure in infants were available.

**Added value of this study**

This study provides the first pharmacokinetic and pharmacodynamic data on oral valganciclovir in infants with severe HIV-associated pneumonia treated empirically for CMV infection. Valganciclovir was administered at 16 mg/kg twice daily. Embedded within the EMPIRICAL randomised clinical trial, it evaluates ganciclovir dosing in a vulnerable population from Africa with high mortality. Our findings confirm that the dosing regimen used in the EMPIRICAL trial results in ganciclovir exposures comparable to those reported in other paediatric populations. Importantly, ganciclovir exposure was not associated with CMV viral load reduction. These findings question the applicability of adult-derived pharmacokinetic targets in this context and support the clinical effectiveness demonstrated in EMPIRICAL trial.

**Implications of all the available evidence**

Taken together, the available evidence supports weight-based valganciclovir dosing at 16 mg/kg twice daily for empirical treatment of CMV in infants with severe HIV-associated pneumonia. This approach is particularly relevant in resource-limited settings, where complex dosing algorithms are difficult to implement. Most children had ganciclovir exposures below the adult target and no clear exposure–response relationship was observed. These findings suggest that lower ganciclovir exposures than the suggested target (AUC_0–12 h_ 40–60 mg∗h/L) may be sufficient in infants.

As the main EMPIRICAL trial demonstrated improved survival with empirical valganciclovir treatment and its results are expected to be incorporated into future international treatment guidelines, these pharmacokinetic data provide important supportive evidence for policy and clinical practice. Future research should focus on defining population-specific ganciclovir pharmacokinetic–pharmacodynamic targets to further refine dosing strategies for CMV treatment in infants.

## Introduction

Cytomegalovirus (CMV) is an ubiquitous viral infection that is transmitted through body fluids, such as saliva, urine, blood, tears, semen, and breast milk.^1^ CMV infection is associated with significant morbidity and mortality among immunocompromised children and adults. In immunosuppressed infants pneumonia is a common manifestation of the active postnatal CMV infection.^1^ CMV seroprevalence varies globally, and Africa stands out with one of the highest prevalence rates, which is estimated to be around 88% in the general population.^2^^,^^3^ In African settings, the majority of children acquire CMV infection during infancy, with mother-to-child transmission predominantly through breastfeeding, being the most common route of infection.^4^^,^^5^ CMV viraemia is commonly detected in infants with pneumonia and meningitis, and poor treatment outcomes are strongly associated with advanced HIV disease.^6^, ^7^, ^8^, ^9^ For infants under 6 months of age, CMV has even been reported to be the second leading cause of death among infants living with HIV with pneumonia, accounting for approximately 10–30% of all deaths.^10^^,^^11^ Despite this, treatment for CMV in children living with HIV remains largely unexplored, due to poor access to CMV diagnosis and treatment in resource-limited settings.^8^^,^^12^

Valganciclovir, a prodrug of ganciclovir, has shown to be an effective, well-tolerated antiviral drug for the treatment and prevention of CMV infection in immunocompromised adults and children.^13^^,^^14^ Furthermore, empirical treatment using valganciclovir was found to have favourable outcomes on the survival of infants with severe HIV-associated pneumonia.^15^ However, its use is commonly associated with adverse events such as anaemia and neutropenia.^14^^,^^16^ The drug is eliminated via glomerular filtration as well as active tubular secretion.^16^

In adult solid organ transplant recipients, ganciclovir is believed to have a narrow therapeutic range (AUC_0–12 h_ 40–60 mg∗h/L) for CMV treatment that has been extrapolated from the pharmacokinetic target for CMV prophylaxis.^17^^,^^18^ Current dosing information for valganciclovir in children is based on studies on CMV treatment for either solid organ transplant patients or treatment of newborns with symptomatic congenital CMV infection.^19^^,^^20^ For the later, valganciclovir is generally administered off-label at a dose of 16 mg/kg body weight twice daily, as it is effective at this dose.^14^^,^^20^ For post-transplant CMV prophylaxis in children, valganciclovir is administered once-daily following the FDA approved dosing regimen based on body surface area (BSA) and estimated glomerular filtration rate (eGFR): Dose (mg/day) = 7 ∗ BSA (m^2^) ∗ eGFR (mL/min/1.73 m^2^).^19^

There is lack of pharmacokinetic data to support valganciclovir dosing for empirical treatment of postnatal CMV, particularly in infants living with HIV who may have altered pharmacokinetics due to malnutrition, diarrhoea and other opportunistic infections.^21^

This study evaluated the pharmacokinetics of valganciclovir administered at a dose of 16 mg/kg twice daily for empirical CMV treatment in infants with HIV with severe pneumonia and examined its association with CMV viraemia reduction and neutropenia. Furthermore, we aimed to identify sources of pharmacokinetic variability of ganciclovir in this specific population and assess the potential impact of using a different dosing regimen based on BSA and eGFR. A better understanding of valganciclovir pharmacokinetics in these vulnerable infants is crucial for optimising antiviral treatment and improving clinical outcomes.

## Methods

### Study population and design

This open-label, single-arm, pharmacokinetic sub-study was nested in the EMPIRICAL randomized controlled trial (NCT03915366), which aims to evaluate whether empirical valganciclovir treatment for cytomegalovirus and rifampicin-based tuberculosis treatment improves the survival of infants with severe HIV-associated pneumonia. The main trial protocol has been published previously.^22^ Inclusion criteria for the main trial were as follows: infants aged between 28 and 365 days with confirmed HIV infection who are diagnosed with pneumonia and meeting admission criteria and requiring parenteral antibiotics following WHO guidelines.^22^ Eligible infants for the main trial were randomly assigned to one of four treatment arms: (1) standard of care (SOC; antibiotics, therapeutic cotrimoxazole, antiretroviral therapy and prednisolone), (2) SOC plus first-line rifampicin-based TB treatment for 6 months, (3) SOC plus valganciclovir at a dose of 16 mg/kg twice daily for 15 days, and (4) SOC plus both TB treatment and valganciclovir. Because of this randomisation process, and since infants in the non-TB treatment arm could still be diagnosed with and treated for TB, approximately half of the included infants received concomitant rifampicin-based TB treatment. As rifampicin is known to induce MRP4 and cause inhibition of OAT1 transporters,^23^^,^^24^ it may interact with the pharmacokinetics of ganciclovir. Hence, co-treatment with rifampicin was accounted for in the statistical analysis.

In the main EMPIRICAL clinical trial, a total of 276 infants were enrolled in the arms receiving valganciclovir. We aimed to pragmatically include the first 100 infants who were enrolled at 13 EMPIRICAL secondary and tertiary hospitals in Mozambique, Uganda, Zambia, and Zimbabwe, in this pharmacokinetic sub-study. Infants weighing less than 3 kg or having an eGFR (Schwartz) of <20 mL/min/1.73 m^2^ were excluded. Data were collected between August 2020 and August 2022. Patients were not involved in the trial design or conduct. Community advisory boards at participating sites were informed about the study and provided advisory support. The pharmacokinetic analyses presented in this study are considered post-hoc analyses of the main clinical trial.

### Procedures

Children received valganciclovir hydrochloride 50 mg/mL powder for oral solution (Roche Ltd.) dosed at 16 mg/kg twice daily for 15 days starting as soon as possible after enrolment. At the day of pharmacokinetic sampling (day 3 after enrolment), the morning valganciclovir dose was administered by study nurses. Blood samples were drawn at 2 and 5 h after drug administration, provided that the infants had received at least 3 doses of valganciclovir to achieve steady state conditions. All infants were hospitalized during the PK visit, and all doses prior to PK sampling were recorded and administered by study nurses. Medication adherence during the two days prior to the pharmacokinetic study visit day was confirmed based on hospital drug administration records. Blood samples were processed within 24 h after sampling and isolated plasma was temporally stored at −80 °C at the recruiting facilities. The samples were then shipped on dry ice to the Department of Pharmacy, Pharmacology & Toxicology, Radboud university medical center, Nijmegen, The Netherlands, for centralized analysis of the samples. A material transfer agreement was in place and approved by relevant authorities in all participating countries. Ganciclovir concentrations were determined using a validated ultra-high performance liquid chromatography (UPLC) assay with a lower and upper limit of quantification of 0.10 mg/L and 30 mg/L, respectively. The assay was validated according to the most recent European Medicines Agency (EMA) guidelines for bioanalytical method validation. The precision of the assay, expressed as coefficient of variation, showed a range of 94.67%–101.7% within runs and 96.33%–100.5% between runs. The assay accuracy was observed to be between 1.79% and 3.70% within runs and 0.00%–1.52% between runs.

Data on demographics, concomitant medication, and CMV viraemia were collected on study visit day 0 and day 15. Full blood count for neutrophil count and Hb was done at 15 days. CMV viral loads and full blood counts were systematically assessed as secondary outcomes of the main clinical trial.

### Statistical analysis

Ganciclovir area-under-curve for the 12 h dosing interval (AUC_0–12 h_) was estimated using the limited sampling equation by Villeneuve et al. (AUC_0–12 h_ = 2.7 ∗ AUC_2–5 h_ + 6).^25^ The combined ganciclovir AUC_0–12 h_ are presented as geometric mean (GM) and inter-subject coefficient of variation (CV%). The proportion of infants with AUC_0–12 h_ within the adult CMV treatment target range (40–60 mg∗h/L) is reported.^17^^,^^18^ The statistical analysis plan is included in Supplementary File 1.

An alternative paediatric valganciclovir dosing regimen is specified in the product label: mg/dose = 3.5 ∗ BSA ∗ eGFR according to the Schwarz formula, capped at 150 mL/min/1.73 m^2^.^26^ To assess the adequacy of this regimen in our study population, we estimated the ganciclovir AUC_0–12h_ assuming hypothetical administration of this dose. The actual ganciclovir AUC_0–12 h_ was proportionally adjusted based on the difference between the administered and calculated doses, assuming first-order pharmacokinetics for valganciclovir.

To explore the relationship between various continuous variables (eGFR, weight-for-length z-score, BSA, and age) and ganciclovir AUC_0–12h_, a Pearson correlation test was performed on log-transformed data. AUC values were log-transformed. To assess the effect of concomitant rifampicin use and sex on ganciclovir AUC_0–12 h_, an unpaired t-test on log-transformed AUC values was used. Other variables were analysed on their original scale. For univariate analyses, a p-value of < 0.05 was considered statistically significant. For covariates correlating with ganciclovir AUC_0–12 h_ with a significance level of p < 0.1, multiple linear regression on log-transformed data was conducted to assess if associations remained significant after correcting for other covariates. The multivariable regression exploratory analysis was performed as post-hoc analyses. Statistical analysis was carried out using IBM® SPSS® Statistics software version 25.

To examine the relationship between ganciclovir AUC_0–12 h_ and reduction in CMV viraemia, linear regression was performed between AUC_0–12 h_ and the logarithmic change in CMV load from day 0 to day 15. Additionally, to evaluate the applicability of the adult AUC_0–12 h_ target in this population, one-way ANOVA was done to compare AUC_0–12 h_ categories (<40, 40–60, >60 mg∗h/L) with the log decrease in CMV load. For safety assessment, one-way ANOVA compared median AUC_0–12 h_ across neutropenia grades, none, grade 1 (0.75–<1.0 ∗ 10^9^ cells/L), grade 2 (0.5–0.749 ∗ 10^9^ cells/L), grade 3 (0.25–0.499 ∗ 10^9^ cells/L), and grade 4 (<0.250 ∗ 10^9^ cells/L), and for anaemia (see Supplementary File 2 for cut-off values for grading). Only children with plasma CMV viral load available at both day 0 and day 15, and children with their neutrophil count and Hb recorded at day 15 were included in the analyses.

### Ethics

The EMPIRICAL trial protocol, including the pharmacokinetic sub-studies, was approved by local ethics committees from all participating countries. All ethical approval numbers are included in Supplementary File 3. Caregivers of the participating infants provided written informed consent for the main study, and an opt-in option was given for inclusion in this specific sub-study. The study consent documents were translated into local languages to ensure clear understanding and informed decision-making.

### Role of the funding source

The funder had no role in the study design, data collection, data analysis, data interpretation, or writing of the report.

## Results

### Population

Eighty-seven out of 98 infants enrolled in this sub-study were included in the analysis. We excluded a total of eleven infants from the analysis: four infants were excluded because samples were drawn at incorrect times after valganciclovir administration, five infants were omitted due to one or two samples falling below the lower limit of quantification (LLOQ), one infant was excluded because the 5hr sample was lost, and one infant was excluded because the child sadly passed away between the 2 hr and 5 hr sampling period. All demographic characteristics are depicted in Table 1.

**Table 1:** Demographic data on the day of pharmacokinetic sampling (study visit day 3).

Demographics	Value
Sex (n (%))	
*Female*	41 (47%)
*Male*	46 (53%)
Age (months)	4.5 (3.2; 7.0)
Weight (kg)	4.5 (4.0; 5.5)
Length (cm)	59 (55; 62)
BSA (m^2^)	0.27 (0.25; 0.31)
WLZ	−1.9 (−3.1; −0.7)
WAZ	−2.6 (−4.7; −1.8)
eGFR∗ (mL/min/1.73 m^2^)	111.6 (69.8; 137.7)
Concomitant TB treatment (n (%))	
*Yes*	42 (48%)
*No*	45 (52%)
Valganciclovir dose per administration (mg)	75 (65; 90)
Valganciclovir dose per kg (mg/kg)	16.0 (15.6; 16.7)

Values are repored as median (IQR) unless specified otherwise. Abbreviations: BSA, body-surface-area; eGFR, estimated glomerular filtration rate; TB, tuberculosis; WAZ, weight-for-age z-score; WLZ, weight-for-height z-score.

### Pharmacokinetics

**Fig. 1:**
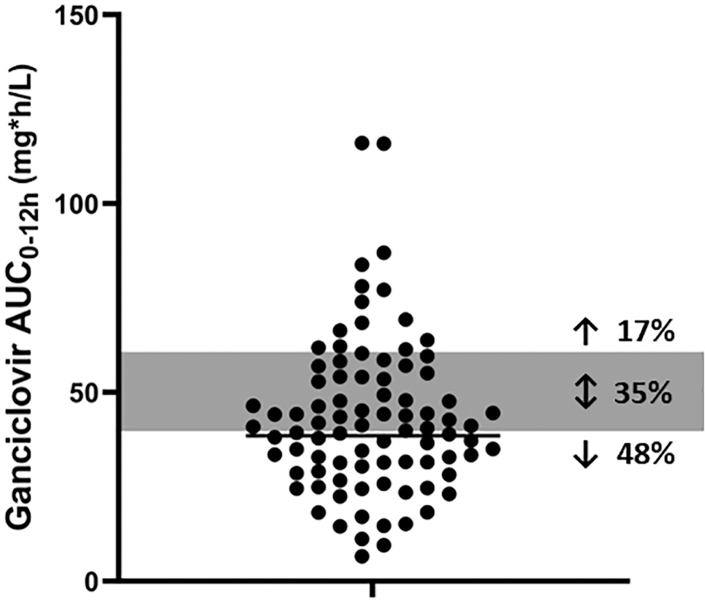
**Individual ganciclovir AUC**_**0–12 h**_**values.** The grey bar represents the target AUC_0–12h_ extrapolated from ganciclovir treatment for CMV disease in adult post-transplant patients. The black line represents the geometric mean AUC_0–12 h_. Abbreviations: AUC, area-under-the-curve Using the BSA/eGFR-based dosing regimen would have resulted in a median (IQR) mg/kg dose of 22.0 (13.2; 28.4) and a GM (CV%) ganciclovir AUC_0–12 h_ of 46.6 (65.5) mg∗h/L (based on proportional scaling), see Table 2.

The GM (CV%) ganciclovir was 38.5 (54.8) mg∗h/L in the total population, with a GM (CV%) C_2 h_ (mg/L) of 4.82 (90.3) mg/L and a C_5 h_ of 2.48 (106.9) mg/L. Overall, 17% of infants had ganciclovir AUC_0–12 h_ above the adult target, 35% fell within the target, and 48% below the target, see Fig. 1 and Table 2.

**Table 2:** Ganciclovir dose, geometric mean AUC_0–12 h_ values, and percentage within the adult AUC_0–12 h_ target for infants within the trial receiving 16 mg/kg, and extrapolated to a valganciclovir mg dose of 3.5 ∗ BSA ∗ eGFR.

	16 mg/kg/dose	3.5∗BSA∗eGFR/dose
Inclusions	87	86^a^
Total valganciclovir dose (mg)	75 (65; 90)	106 (64; 138)
Valganciclovir dose per kg (mg/kg)	16.0 (15.6; 16.7)	22.0 (13.2; 28.4)
GM ganciclovir AUC_0–12 h_ (mg∗h/L; CV%)	38.5 (54.8)	46.6 (65.5)^b^
% within AUC_0–12 h_ target		
*<40 mg∗h/L*	48%	36%^b^
*40–60 mg∗h/L*	35%	27%^b^
*>60 mg∗h/L*	17%	37%^b^

Values are repored as median (IQR) unless specified otherwise.

Abbreviations: AUC, area-under-the-curve; BSA, body-surface-area; eGFR, estimated glomerular filtration rate; GM, geometric mean.

a For one infant, the eGFR was unknown. This infant was therefore excluded from this analysis.

b These data are proportionally scaled from the actual AUC values using the difference in dose upon using the alternative dosing regimen.

**Fig. 2:**
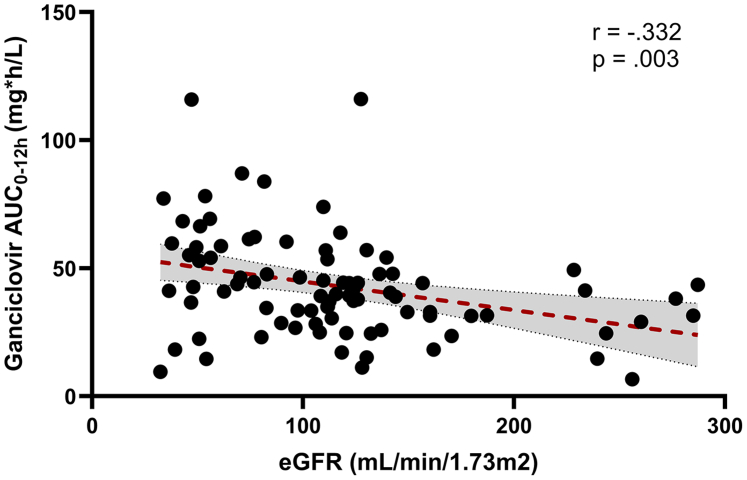
**Individual ganciclovir AUC**_**0–12 h**_**values plotted against eGFR.** Abbreviations: AUC, area-under-the-curve; eGFR, estimated glomerular filtration rate (Schwartz method).

Concomitant use of rifampicin-based TB treatment did not affect ganciclovir AUC_0–12 h_ (geometric mean ration (95% confidence interval): 1.00 (0.80–1.24); Supplementary File 4) nor did sex (0.94 (0.75–1.17). On the other hand, there was a moderately negative correlation between age (r (85) = −0.332, p = 0.002) and eGFR (r (84) = −0.312, p = 0.003; Fig. 2) with log-transformed ganciclovir AUC_0–12 h_. No statistically significant correlation was found between weight-for-length z-score (r (85) = 00.194, p = 0.072) and BSA (r (85) = −0.196, p = 0.068) with ganciclovir AUC_0–12 h_. After multivariate analysis, including age, eGFR, weight-for-length z-score, and BSA, only increasing eGFR was significantly associated with decreased ganciclovir AUC_0–12 h_ (p = 0.028). Univariate and multivariate statistical test details are included in Supplementary File 5).

### Pharmacodynamic analysis

**Fig. 3:**
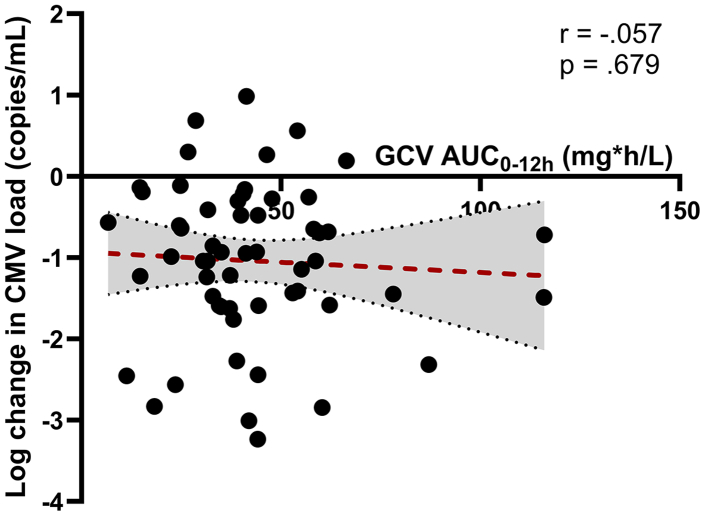
**Individual ganciclovir AUC**_**0–12 h**_**values plotted against log decrease in CMV viraemia.** Abbreviations: AUC, area-under-the-curve; GCV, ganciclovir.

**Fig. 4:**
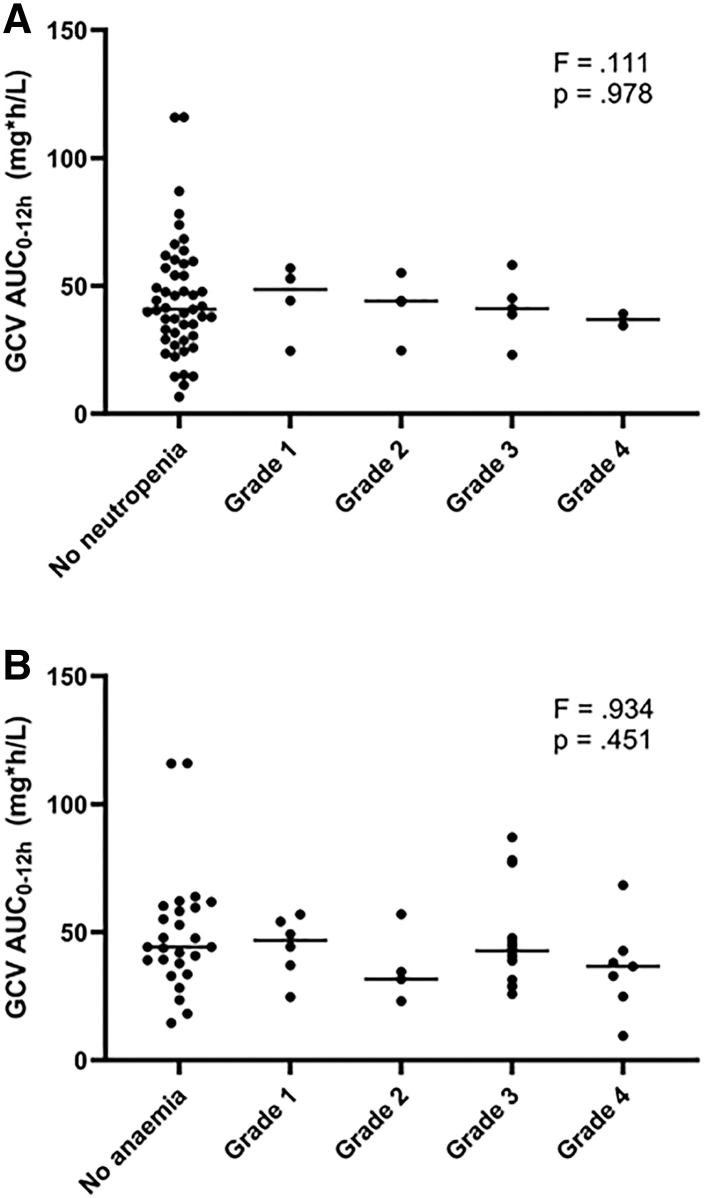
**Individual ganciclovir AUC**_**0–12 h**_**values per neutropenia grade (A) and per anaemia grade (B).** Abbreviations: AUC, area-under-the-curve; GCV, ganciclovir.

The relationship between ganciclovir AUC_0–12 h_ and log decrease in CMV viraemia is shown in Fig. 3 and with grades of neutropenia and anaemia in Fig. 4. Individual CMV viral load data at baseline and on day 15 are included in Supplementary File 6. The median CMV viral load decrease was 1.04 (IQR 0.43–2.29) log copies/ml in 55 infants for whom viral load data were available on day 0 and 15. The linear regression analysis revealed no significant association between ganciclovir AUC_0–12 h_ and the decrease in CMV load from day 0 to day 15 of treatment, F (1, 54) = 0.174, p = 0.679. Similarly, the one-way ANOVA indicated no significant differences in CMV load reduction across the AUC_0–12 h_ groups, F (2, 53) = 0.65, p = 0.529.

Of 65 infants with a neutrophil count at day 15 of the study, 8 had Grade 1; 9 had Grade 2; 9 had Grade 3; and 2 had Grade 4 neutropenia; and 37 were not neutropenic. The one-way ANOVA test showed no significant differences in ganciclovir AUC_0–12 h_ across the various neutropenia grades, F (4, 60) = 0.111, p = 0.978.

Of 60 infants with an Hb measurement at day 15 of the study, 6 had Grade 1; 5 had Grade 2; 16 had Grade 3; 7 had Grade 4 anaemia; and 26 were not anaemic. The one-way ANOVA test showed no significant differences in ganciclovir AUC_0–12 h_ across the various anaemia grades, F (4, 55) = 0.934, p = 0.451.

## Discussion

This is the first study to report on ganciclovir pharmacokinetics in a vulnerable population of hospitalised infants with severe HIV-associated pneumonia of whom many were malnourished receiving oral valganciclovir for empirical CMV treatment. Interpatient variability of ganciclovir AUC was large in our population and predefined adult CMV treatment targets were not achieved in almost 50% of the infants upon administration of valganciclovir oral solution 16 mg/kg/dose twice daily. Nevertheless, lower ganciclovir exposure did not appear to affect the magnitude of CMV viraemia reduction in our cohort. Notably, in the main EMPIRICAL trial, valganciclovir improved survival in infants with severe HIV-associated pneumonia.^15^^,^^27^

Similar to findings in previous research, we observed that ganciclovir exposure tended to be lower and more variable in infants compared to adults after administration of recommended dosages.^18^^,^^28^^,^^29^ A pharmacokinetic study in a paediatric transplant population aged between 7 and 42 months using weight-based valganciclovir dosages comparable to the dosages used in our study (14–16 mg/kg/dose) demonstrated 11.5%, 50%, and 38.5% of children to have ganciclovir levels above, within and below the AUC 40–60 mg∗h/L target, respectively.^25^ In our study, we found comparable percentages of children above, within and below the target (17%, 35%, and 48%, respectively). Furthermore, Launay et al. reported a median 20 mg/kg dose-normalized ganciclovir AUC of 37.6 mg∗h/L in 10 children (8 months–13.1 years old).^30^ This is similar to the GM AUC reported in our study after administration of 16 mg/kg (38.5 mg∗h/L) Additionally, the GM C_2 h_ of 4.82 mg/L found in our study was slightly higher compared to the median C_2 h_ of 3.36 mg/L reported in a population of 13 infants between 30 and 60 days old treated for congenital CMV.^31^

In the context of post-transplant prophylaxis or treatment, valganciclovir dosing in children is often done following a complex algorithm, including eGFR and BSA (valganciclovir dose per gift (mg) = 3.5 × BSA × eGFR according to the Schwarz formula), that generally results in relatively higher dosages for children with normal kidney function compared to the mg/kg dosing.^32^ More children seem to achieve the adult target AUC_0–12 h_ (40–60 mg∗h/L) using this dosing approach, particularly in younger patients.^26^^,^^32^ However, in our study population, this dosing approach would have resulted in many children having high ganciclovir exposures with 37% having an AUC_0–12 h_ of >60 mg∗h/L and only 27% within the adult target, and a larger inter-individual variation in AUC_0–12 h_, based on proportional scaling. Moreover, the practical implementation of such a dosing strategy may be challenging in resource-limited settings.

Reaching a consensus on appropriate pharmacokinetic targets remains essential before assessment of adequacy of dosing strategies. In our study, despite the wide range in ganciclovir exposure seen, we observed no correlation between ganciclovir exposure and either CMV viral load reduction. These findings suggest that the adult AUC_0–12 h_ target may not be applicable in our patient cohort. Notably, the main EMPIRICAL trial demonstrated improved survival in infants receiving valganciclovir 16 mg/kg twice daily for two weeks. This is in line with other clinical trials that reported favourable outcomes in children with congenital CMV despite lower ganciclovir exposures compared to adults.^14^^,^^33^ Furthermore, various pharmacokinetic studies have shown favourable virological outcomes in infants with exposures below the adult target.^17^^,^^18^^,^^25^^,^^34^^,^^35^

This raises the question whether the pharmacokinetic target that was established in adult transplant patients is also applicable for paediatric patients and to other causes of CMV disease, such as congenital CMV and HIV-associated CMV pneumonia.^36^ For treatment of congenital CMV infection, a different ganciclovir pharmacokinetic target has been suggested by Kimberlin et al. (AUC_0–12 h_ 20–55 mg∗h/L).^20^ These cut-off values represent the 10th–90th percentile around the median ganciclovir AUC_0–12 h_ (27 mg∗h/L) observed after intravenous administration of 6 mg/kg ganciclovir to neonates in a previous pharmacokinetic study.^37^ In our study, 58 out of 87 infants (67%) had a ganciclovir AUC_0–12 h_ between 20 and 50 mg∗h/L. However, it is essential to note that this target was not established through a formal exposure-response analysis. To better understand optimal valganciclovir dosing for CMV pneumonia in infants living with HIV, further pharmacokinetic/pharmacodynamic studies are needed to establish the exposure–response relationship for both efficacy and safety in this population.

As ganciclovir is renally excreted, it is not surprising that ganciclovir exposure increased with decreasing eGFR in our study. Previous studies have found similar relationships between eGFR and ganciclovir levels in children.^18^^,^^19^^,^^38^ Renal insufficiency at time of recruitment in our cohort is believed to be driven mainly by shock and severe illness. Furthermore, children living with HIV are more likely to have impaired kidney function, particularly those with advanced HIV disease.^39^ Additionally, antibiotics for the treatment of severe pneumonia, such as cotrimoxazole and aminoglycosides could cause kidney damage.^40^ Therefore, we recommend regular monitoring of eGFR in children using valganciclovir and adjusting the dose accordingly.^16^

This study had several limitations that need to be considered when interpreting the study results. Firstly, we used a limited sampling method to reduce the burden of the study for the children that were generally very sick during sampling. It should be noted that the limited sampling method was originally developed and validated based on a population of young children (aged 6 months to 3 years) without HIV receiving a valganciclovir dose similar to our study. As the limited sampling method was not validated for infants living with HIV aged 1–12 months, this could introduce some uncertainty around the accuracy of the estimated AUCs due to potential variations in the absorption profile of valganciclovir resulting from uncontrolled HIV infection and the severe illness of the children enrolled in this study. Secondly, we measured creatinine for eGFR determination upon inclusion in the main trial, while the blood samples used for this study were drawn on day 3 of the main study. Furthermore, the different sites used different methods (i.e. point-of-care tests and Jaffe method) to measure creatinine, which could have contributed to discrepancies in the eGFR results. Despite these limitations, the clear correlation between eGFR and ganciclovir exposure supports the validity of the results, consistent with renal clearance of ganciclovir. While the total sample size included 87 eligible infants, fewer were available for the exposure–response analyses (55–65 infants) due to missing CMV VL, Hb, and neutrophil data. In addition, the incidence of neutropenia was low. Together, these factors reduced the statistical power of the one-way ANOVA, and the absence of a statistically significant association between ganciclovir exposure and neutropenia should therefore be interpreted with caution.

In conclusion, although approximately two-thirds of participants did not achieve the extrapolated adult pharmacokinetic target when receiving valganciclovir at 16 mg/kg/dose twice daily, the pharmacokinetic results were comparable to other studies in infants. Furthermore, ganciclovir exposure did not correlate with CMV plasma viral load reduction, suggesting lower exposures did not affect treatment efficacy in the EMPIRICAL study. Future research is needed better to understand valganciclovir pharmacokinetic/pharmacodynamic relationships in children.

## Contributors

TJ, AC, DB, and PR conceived the study. Data analysis was done by TJ and VMum with support from SD and AC (statistics/pharmacokinetics), and JT, FE and MB (CMV viral load). Data collection was done by VMum, LB, AP, LK, MC, BN, JB, KC, SM, NM, MB, NN, JS, DN, HM, CC, WCB, and VMus. TJ and VMum wrote the first draft of the manuscript and all other authors made essential revisions to and approved the final manuscript. TJ, VMum, SD, CM, AC, DB, and PR had full access to all data in the study and final responsibility for the decision to submit for publication.

## Data sharing statement

The data that support the findings of this study are available from the corresponding authors upon reasonable request.

## Declaration of generative AI and AI-assisted technologies in the manuscript preparation process

During the preparation of this work, the author(s) used ChatGPT (GPT-4o, OpenAI) solely to improve the readability and clarity of the language. After using this tool, the author(s) reviewed and edited the content as needed and take(s) full responsibility for the content of the published article.

## Declaration of interests

The authors declare no competing interests.
