# Supplementary material for: Pharmacokinetics and pharmacodynamics of valganciclovir in infants with severe HIV-associated pneumonia in Africa: a sub-study of the EMPIRICAL randomised controlled trial
Source: eClinicalMedicine. 2026 Apr 13;94:103899. doi: 10.1016/j.eclinm.2026.103899 (PMC13092656; doi:10.1016/j.eclinm.2026.103899)
Supplement: Supplementary Files [file mmc1.docx]

**Supplementary file 1: statistical analysis plan**

**EMPIRICAL**

**Statistical Analysis Plan PK-3 (valganciclovir) studies**

**Version 1.1**

19^th^ June 2024

**Pharmacokinetics and pharmacodynamics of ganciclovir upon administration of oral valganciclovir for empirical treatment of cytomegalovirus infection in infants with severe HIV-associated pneumonia**

Revision history

| **Version** | **Date** | **Reason for revision** |
| --- | --- | --- |
| 1.0 | 13/02/2022 | Document created |
| 1.1 | 19/06/2024 | Added PK/PD analysis and alternative dosing analysis; recommendation by the trial steering committee. |

# Introduction PK study

This document includes the plan for the pharmacokinetic (PK) and pharmacodynamic (PD) analysis of (val)ganciclovir. The aim of the EMPIRICAL PK-3 substudy is to evaluate adequacy of the used valganciclovir dosing regimen (16 mg/kg twice daily (BID)), to identify sources of pharmacokinetic variability, and to understand rates of efficacy and toxicity of empirical valganciclovir treatment.

The objectives of this PK substudy were to:

- To evaluate the used valganciclovir dosing regimen
- To identify sources of pharmacokinetic variability
- To understand rates of efficacy and toxicity of empirical valganciclovir treatment

# Participants

### 2.1 Sample size

We plan to recruit around 100 infants aged 1-12 months that have enrolled in the main EMPIRICAL trial and receiving valganciclovir. Additional exclusion criteria include weighing less than 3kgs and having an estimated glomerular filtration of <20 ml/min/1.73 m^2^. Children are enrolled at EMPIRICAL clinical sites in Mozambique (UEM and Manhiça), Uganda, Zambia, and Zimbabwe. Caretakers are asked to participate in this PK sub-study by a direct question in the main ICF of the EMPIRICAL study.

Children were administered 16 mg/kg valganciclovir BID for 15 days after being randomized to one of the valganciclovir arms in the EMPIRICAL trial. Blood samples for this PK substudy were drawn at 2 and 5 hours after drug administration during the Day 3 visit of the main trial; infants should have received at least 3 gifts of valganciclovir prior to the PK visit.

### 2.2 Exclusion of subjects

Subjects/samples can be excluded from the statistical analysis of pharmacokinetics on a case-by-case basis if no reliable pharmacokinetic parameters can be determined. A subject can be excluded from data evaluation if justified by circumstances:

- Vomiting within 0.5 to 4 hours after drug administration or severe diarrhoea; PK data for these children will be evaluated on a case-by-case basis.
- If the actual time of blood drawing deviates ±10% from the scheduled blood sampling time.
- If one of the two samples is missing or if it is unclear which sample is drawn at t=2hrs and t=5hrs.
- Samples are not processed within time limits for laboratory procedures stated in MOP (24 hours after sampling).

The decision for exclusion will ideally be made before bioanalysis. When a decision is made (before or after bioanalysis), it will be clearly documented and justified. Before bioanalysis, eCRFs will be reviewed and automated queries are implemented to confirm PK visits were conducted according to the instructions in the protocol and SOP. At a minimum, data will checked for the circumstances described above, and for: plausibility of weight and height on PK against the most recent weight and height on main trial follow up form; valganciclovir dose; sampling times; actual versus scheduled – checking for disagreements and missing values; food intake around valganciclovir administration. Excluded subjects will be documented.

# Descriptive data

Data on demographics, treatment compliance, concomitant medication, CMV viral loads, and safety has been recorded for all the included subjects in the main EMPIRICAL trial CRFs. Demographics at the first PK assessment day (including age, sex, weight, eGFR, weight-for-height, weight-for-age, body-surface area (BSA), and dosage (mg/kg body weight)) will be listed. Safety and efficacy of valganciclovir will be evaluated at EMPIRICAL trial visits (with extra visits where necessary) and derived from main trial data.

# Pharmacokinetic data

PK samples for valganciclovir will be drawn at t=2 and 5 hours after dosing. Ganciclovir area-under-curve for the 12 hour dosing interval (AUC_0-12h_) will be estimated using the limited sampling equation by Villeneuve et al (AUC_0-12h_ = 2.7 * ((C_2h_ + C_5h_)/2*3) + 6).(1) Geometric Means and % coefficient of variation will be calculated for the estimated AUC of ganciclovir. The results will be compared to published data in children.

Statistical analysis will be carried out using IBM^®^ SPSS^®^ Statistics software version 25. Raw concentration data input in SPSS will be QCed by a second individual.

### 4.1 How to handle anomalous concentration values

Individual concentrations considered to be anomalous will be excluded from the pharmacokinetic analysis and median and mean profiles; such anomalous values (e.g. not evaluable PK value or profile) will be identified in the data listings of the publication. Anomalous values are those that are inconsistent with known or expected pharmacokinetic behaviour of the drug. Clear justification must be provided in the report for exclusion of any data.

### 4.2 Deviations from scheduled sampling times

Actual sampling times may differ from the scheduled (planned) times. When calculating individual PK parameters PK profiles, the scheduled sampling times will be used as we apply a limited sampling strategy that is validated for those timepoints. The individual sampling time deviations should not deviate too much from the scheduled sampling time as this may affect the validity of AUC_0-12h_ calculations. To account for possible large deviations, the acceptance range for sampling time deviations is set as a percentage of the sampling time. Samples collected outside that acceptance range should be excluded from the calculation of descriptive statistics of the concentrations per time point (mean, standard deviation, coefficient of variation, etc.) and flagged. The acceptance range is set at ±15% deviation from the nominal time.

### 4.4 Pharmacokinetic parameters valganciclovir

#### AUC over a dosing interval

The AUC_0-12h_ will be calculated using the limited sampling equation by Villeneuve et al (AUC_0-12h_ = 2.7 * AUC_2-5h_ + 6). AUC will be reported as amount*time/volume (e.g., mg*h/L).

### 4.5 Descriptive and inferential statistics

We will present the individual and combined ganciclovir AUC_0-12h_ as geometric mean and inter-subject coefficient of variation (CV%). To investigate the relationship between various covariates and ganciclovir concentration, we will plot individual ganciclovir AUC_0-12h_ against eGFR, weight-for-age / weight-for-height, BSA, age, and concomitant rifampicin-based TB treatment. Spearman's rank test will be used to assess the correlation between continuous variables and ganciclovir AUC_0-12h_. For the dichotomous variable (concomitant rifampicin-based TB treatment) we will calculate geometric mean ratio by comparing ganciclovir AUC_0-12h_ in the children receiving rifampicin versus those who don’t groups using an unpaired t-test on log-transformed data.

To evaluate adequacy of dosing, our results will be compared with literature data for children in the same age group receiving a similar dose (1-4). In addition, the proportion of infants with AUC_0-12h_ within the suggested target range for CMV treatment in adults (40-60 mg*h/L) will be reported (3-5). Then we will estimate the AUC_0-12h_ if another registered valganciclovir dosing regimen would have been administered; mg/day = 7 * BSA * eGFR according to the Schwarz formula, capped at 150 mL/min/1.73 m^2^(6). The actual AUC_0-12h_ will be proportionally increased or decreased with the difference in calculated dose as valganciclovir yields first order pharmacokinetics.

Only infants with baseline and day 15 data on CMV viral load in blood available in the main trial will be included in the assessment of the relationship between ganciclovir exposure and efficacy. We will plot the CMV viral load decay over 15 days against ganciclovir AUC_0-12h_ for all individuals to evaluate if there is a relationship between ganciclovir exposure and CMV load decrease and use a linear regression to evaluate the association between AUC_0-12h_ and CMV viral decay in blood. Then, we will assess the log decrease in blood CMV viral load in infants that are below the target (<40 mg*h/L), within the target (40-60 mg*h/L), and above the target (>60 mg*h/L). A one-way ANOVA will be done to statistically test for differences in log decrease in plasma CMV viral load between the various AUC_0-12h_ categories.

To assess the PK/PD relationship between ganciclovir and its toxicity, one-way ANOVA will be done to compare median AUC_0-12h_ across neutropenia grades, none, grade 1 (0.75 – <1.0 * 10^9^ cells/L), grade 2 (0.5 – 0.749 * 10^9^ cells/L), grade 3 (0.25 – 0.499 * 10^9^ cells/L), and grade 4 (<0.250 * 10^9^ cells/L).

**Supplementary file 2: Grading system for anaemia**

| **Hemoglobin** *(g/dL; mmol/L)* | **Grade 1** *Mild* | **Grade 2** *Moderate* | **Grade 3** *Severe* | **Grade 4** *Potentially life-threatening* |
| --- | --- | --- | --- | --- |
| 57 days of age to < 13 years of age (male and female) | 9.5 to 10.4  5.88 to 6.48 | 8.5 to < 9.5  5.25 to < 5.88 | 6.5 to < 8.5  4.03 to < 5.25 | < 6.5  < 4.03 |
| 36 to 56 days of age (male and female) | 8.5 to 9.6  5.26 to 5.99 | 7.0 to < 8.5  4.32 to < 5.26 | 6.0 to < 7.0  3.72 to < 4.32 | < 6.0  < 3.72 |
| 22 to 35 days of age (male and female) | 9.5 to 11.0  5.88 to 6.86 | 8.0 to < 9.5  4.94 to < 5.88 | 6.7 to < 8.0  4.15 to < 4.94 | < 6.7  < 4.15 |

**Supplementary file 4: Ethical approval numbers**

| **Country** | **Committee** | **Reference number** |
| --- | --- | --- |
| Zimbabwe | Joint Research Ethics Committee (JREC) | JREC/169/19 |
| Zimbabwe | Medical Research Council of Zimbabwe | MRCZ/A/2511 |
| Zimbabwe | Research Council of Zimbabwe | No 05065; No 05396 |
| Zimbabwe | Medicines Control Authority of Zimbabwe | B/279/5/202/2025 |
| Uganda | Makerere University School of Medicine Research and Ethics Committee | REC REF No. 2019-115 |
| Uganda | National Drug Authority | CTC 0119/2024 |
| Zambia | Biomedical Research Ethics Committee, University of Zambia | 059-2019 |
| Mozambique | Comité Nacional de Bioética para a Saúde (CNBS) | Ref: 335/CNBS/24 |

**Supplementary file 4: Boxplot ganciclovir AUC_0-12h_ in infants receiving concomitant TB treatment**

Boxplots representing ganciclovir AUC_0-12h_ in infants receiving concomitant TB treatment and those not receiving TB treatment. The boxes and whiskers represent the interquartile range (IQR) of the data distribution. Abbreviations: AUC, area-under-the-curve; TB, tuberculosis.


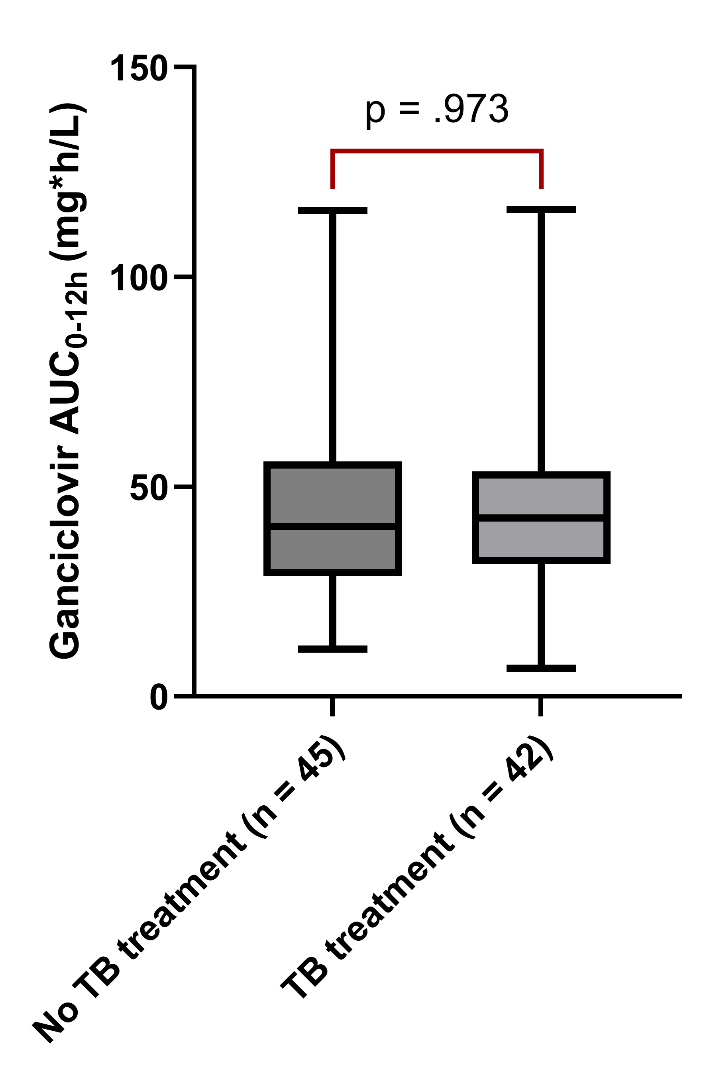


**Supplementary file 5: Results covariate analyses**

Pearson correlation between covariates and log-transformed ganciclovir AUC_0-12h_. p<.05 was considered statistically significant (*).
Abbreviations: BSA, body surface area; eGFR, estimated glomerular filtration rate; WLZ, weight-for-length z-score.

| **Covariate** | **R**^2^ | **p-value** |
| --- | --- | --- |
| **Age (months)** | -0.33 | 0.002* |
| **eGFR** | -0.31 | 0.003* |
| **WLZ** | 0.19 | 0.072 |
| **BSA** | -0.20 | 0.068 |

Geometric mean ratios (B) for univariate as well as multivariate analysis including exponentiated 95% confidence interval and p-values. All outcomes with p<.1 were included in the multivariate analysis. Multivariate analysis: linear regression between covariates identified in univariate analysis and log-transformed AUC_0-12h_ values. p<.05 was considered statistically significant (*).
Abbreviations: CI, confidence interval; BSA, body surface area; eGFR, estimated glomerular filtration rate; GMR, geometric mean ratio; WLZ, weight-for-length z-score.

|  | **Univariate analysis** | | | **Multivariate analysis** | | |
| --- | --- | --- | --- | --- | --- | --- |
| **Covariate** | **Univariate GMR** | **95% CI** | **p-value** | **Adjusted GMR** | **95% CI** | **p-value** |
| **Age (month)** | 0.93 | 0.89 – 0.97 | 0.002* | 0.96 | 0.91 – 1.02 | 0.221 |
| **eGFR (ml/min)** | 1.00 | 1.00 – 1.00 | 0.003* | 1.00 | 1.00 – 1.00 | 0.028* |
| **WLZ** | 1.06 | 1.00 – 1.12 | 0.072 | 1.05 | 0.98 – 1.12 | 0.187 |
| **BSA** | 0.11 | 0.01 – 1.19 | 0.068 | 0.44 | 0.01 – 12.76 | 0.625 |
| **Sex (male vs female)** | 0.94 | 0.75 – 1.17 | 0.571 | — | — | — |
| **Rifampicin use** | 1.00 | 0.80 – 1.24 | 0.973 | — | — | — |

**Supplementary file 6: Individual CMV viral load data**

Individual plasma CMV viral load data at baseline and on Day 15. The Y-axis presents the viral load in copies/mL of plasma. The lower limit of quantification was 69 copies/mL.


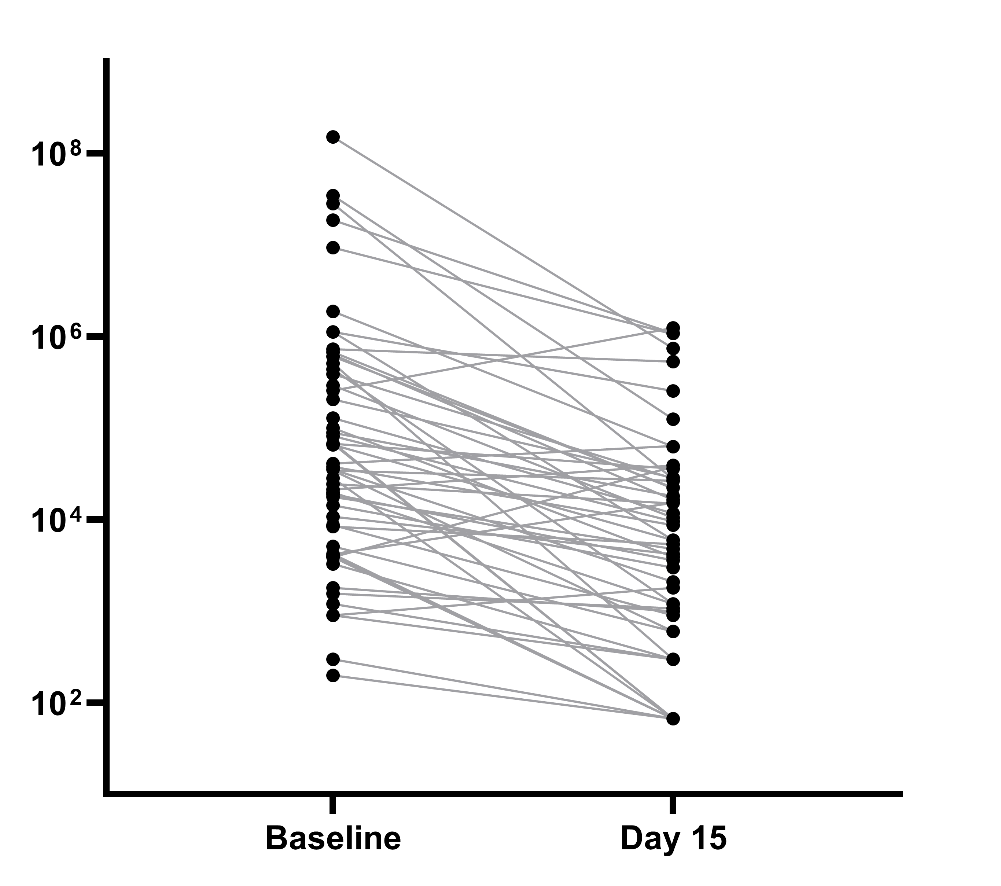


**References**

1. Villeneuve D, Brothers A, Harvey E, Kemna M, Law Y, Nemeth T, Gantt S. Valganciclovir dosing using area under the curve calculations in pediatric solid organ transplant recipients. Pediatr Transplant. 2013;17(1):80-5.

2. Pescovitz MD, Ettenger RB, Strife CF, Sherbotie JR, Thomas SE, McDiarmid S, et al. Pharmacokinetics of oral valganciclovir solution and intravenous ganciclovir in pediatric renal and liver transplant recipients. Transpl Infect Dis. 2010;12(3):195-203.

3. Kimberlin DW, Acosta EP, Sánchez PJ, Sood S, Agrawal V, Homans J, et al. Pharmacokinetic and pharmacodynamic assessment of oral valganciclovir in the treatment of symptomatic congenital cytomegalovirus disease. J Infect Dis. 2008;197(6):836-45.

4. Stockmann C, Roberts JK, Knackstedt ED, Spigarelli MG, Sherwin CM. Clinical pharmacokinetics and pharmacodynamics of ganciclovir and valganciclovir in children with cytomegalovirus infection. Expert Opin Drug Metab Toxicol. 2015;11(2):205-19.

5. Wiltshire H, Paya CV, Pescovitz MD, Humar A, Dominguez E, Washburn K, et al. Pharmacodynamics of oral ganciclovir and valganciclovir in solid organ transplant recipients. Transplantation. 2005;79(11):1477-83.

6. Jorga K, Reigner B, Chavanne C, Alvaro G, Frey N. Pediatric Dosing of Ganciclovir and Valganciclovir: How Model-Based Simulations Can Prevent Underexposure and Potential Treatment Failure. CPT Pharmacometrics Syst Pharmacol. 2019;8(3):167-76.
